# Structural Optimization Design of Magnetoelectric Thin-Film Antenna for Bandwidth and Radiation Enhancement

**DOI:** 10.3390/mi15070810

**Published:** 2024-06-21

**Authors:** Xiangyang Li, Pengchao Zhao, Guangyuan Wang, Na Li, Yiqun Zhang

**Affiliations:** Key Laboratory of Electronic Equipment Structure Design, Ministry of Education, Xidian University, Xi’an 710071, China; 21041110080@stu.xidian.edu.cn (X.L.); pczhao_1@stu.xidian.edu.cn (P.Z.); 13033984579@163.com (G.W.); yiqunzhang@xidian.edu.cn (Y.Z.)

**Keywords:** antenna miniaturization, magnetoelectric antenna, structural optimization, bandwidth, radiation performance

## Abstract

The acoustically actuated nanomechanical magnetoelectric (ME) antennas represent a promising new technology that can significantly reduce antenna size by 1–2 orders of magnitude compared to traditional antennas. However, current ME antennas face challenges such as low antenna gain and narrow operating bandwidth, limiting their engineering applications. In this paper, we enhance the bandwidth and radiation performance of ME antennas through structural optimization, leveraging theoretical analysis and numerical simulations. Our findings indicate that optimizing the inner diameter of the ring-shaped ME antenna can elevate the average stress of the magnetic layer, leading to improved radiation performance and bandwidth compared to circular ME antennas. We establish an optimization model for the radiation performance of the ME antenna and conduct shape optimization simulations using COMSOL Multiphysics. The results of the Multiphysics field optimization align with the stress concentration theory, demonstrating a strong correlation between the radiation performance and bandwidth of the ME antenna with the average stress of the magnetic film. The resonant frequency in the thickness vibration mode is determined to be 170 MHz. Furthermore, shape optimization can enhance the bandwidth by up to 104% compared to circular ME antenna structures of the same size.

## 1. Introduction

Antenna is a tool for receiving and transmitting electromagnetic (EM) waves, is an important part of modern communication systems, and is widely used in smart phones, smart watches, laptops and other devices. One of the most important challenges in the field of antenna research is the miniaturization of its size [1,2,3]. Traditional electric small antenna depends on EM oscillation. The size of the shortest antenna cannot be less than 1/10 of the EM wavelength; otherwise, the radiation and reception of EM waves become ineffective. This principle fundamentally limits the miniaturization of the antenna. Therefore, it becomes crucial to explore new mechanisms for EM wave reception and new mechanisms for the miniaturization of antennas.

In recent years, the concept of mechanical antennas, whose dimensions do not depend on the wavelength of EM waves, has been proposed and has attracted the attention of researchers [4,5]. ME antenna as a kind of mechanical antenna, based on the principle of the ME coupling effect, consists of an ME-composite-laminated structure. Yao et al. proposed for the first time the concept of using piezoelectric and magnetostrictive layers as antenna radiation, and used finite difference time-domain (FDTD) method to model sound waves and EM radiation [4,6]. Nan et al. firstly reported ME antennas and conducted experimental tests [7]. Xu et al. analyzed the converse ME effect and EM radiation of ME-laminated structures, and the results showed that the radiation of ME antennas is an ideal magnetic dipole model [8]. In 2021, Sun et al. developed an ME antenna utilizing an SMR structure, achieving a center frequency of 1.575 GHz through innovative material and structural configurations. This design yielded a −5 dB bandwidth of 42 MHz, along with a fractional bandwidth of 2.6% [9]. In 2022, Li et al. used a Mason model to design a two-layer back-cavity-type ME antenna and used the COMSOL Multiphysics V6.0 to carry out ME performance simulation. The authors finally manufactured an ME antenna operating at 2.45 GHz with a gain of −15.59 dB [10]. Shi et al. proposed a multiphysical field coupling model considering nonlinear magnetostrictive models to model ME antennas [11]. However, the ME antenna still has issues with narrow bandwidth and low gain [1,12,13,14].

Nan et al. introduced the FBAR ME antenna, boasting a center frequency of 2.53 GHz and a −5 dB threshold based on S11, with a bandwidth of 75 MHz and a fractional bandwidth of 2.96% [7]. In 2019, Nikitin et al. conducted simulations on an ME antenna featuring a composition of ferromagnetic yttrium iron garnet and lead zirconate titanate film. The simulated results revealed a center frequency of 2.8 GHz, accompanied by an approximately 73.3 MHz −5 dB bandwidth and a fractional bandwidth of 2.6% [15]. Zaeimbashi et al. made strides in 2021 by unveiling three ME antennas designed in parallel, each boasting a center frequency of 2.51 GHz. These designs exhibited a −5 dB bandwidth of 60 MHz and a fractional bandwidth of 2.39% [14]. Yun et al. proposed a bandwidth-enhanced ME antenna made of Mo/AlN/FeGa sandwich layers composed of three different resonant regions; the antenna achieves a fractional bandwidth of 2.7% (−3 dB) while maintaining the advantage of small size [16]. In the same year, they manufactured and demonstrated an ME antenna driven by a high-overtone bulk-acoustic resonator (HBAR), which has a floating potential structure (FPA). Compared with directly grounded HBAR-ME antennas, FPA can significantly improve the gain and radiation efficiency of HBAR-ME antennas by over 10 dB [17]. In 2023, Shi et al. proposed a new acoustic-actuated antenna using embedded ME composites to improve the radiation of ME antennas through the enhanced strain transfer at the interfaces between different phases [18]. Jin et al. proposed a microbridge structure and array connection method for low-frequency thin-film ME antennas to address the problems of narrow working bandwidths and the weak radiation intensity of ME antennas, and verified them through experiments [19]. Luo et al. utilizes parallel and series antenna array topology to achieve a profound gain and radiation efficiency enhancement without degrading impedance mismatch and quality factor of ME resonators [20]. Dong et al. proposed an ME antenna array consisting of three units, which are constructed in a sandwich stack. The −3 dB operating bandwidth of 152.4–172.8 KHz is achieved, and the relative bandwidth is 12.5% [21].

In summary, current research on ME antennas based on thin-film bulk-acoustic resonators (FBARs) has mainly focused on operations in the GHz range, while relatively little attention has been paid to low-frequency variants below 200 MHz. Particularly lacking is the exploration of enhancing ME antenna bandwidth through structural design considerations. This paper aims to improve the bandwidth and radiation performance of ME antenna through structural optimization. We employ mathematical models and the Multiphysics field simulation software to optimize the internal radius of the circular ME antenna. Our aim is to enhance the radiation power of the antenna and determine the most optimal internal radius. The simulation results align with the predictions of stress concentration theory, demonstrating a consistent trend.

## 2. Theory and Simulation of Me Antenna

**Figure 1:**
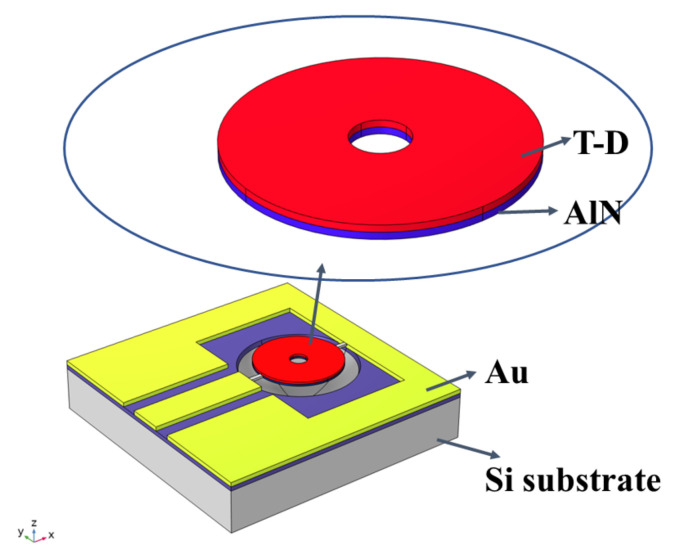
A 3D schematic diagram of ring-shaped ME antenna on released Si substrate.

The 3D structure of a ring-shaped ME antenna is shown in Figure 1, where the illustration magnifies the core structure of an ME-laminated film. The composite magnetostrictive film is used as the core film stack of the antenna, and the piezoelectric layer below it generates acoustic excitation. Since the thickness of the electrode layer is very thin compared with the piezoelectric layer and magnetic layer, we regard the electrode layer as a finitely thin plane.

**Figure 2:**
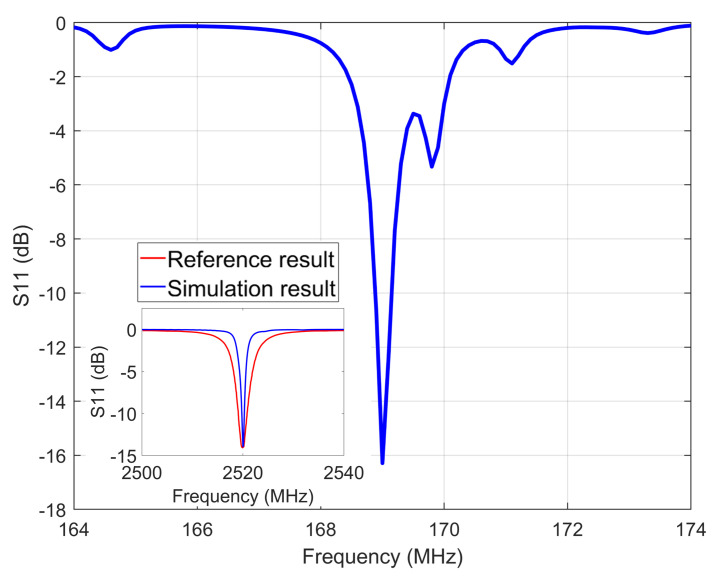
Simulated reflection coefficient S11 of the ME antenna.

In this paper, we demonstrate the ME antenna operating at HF frequencies based on the strong ME coupling EM and bulk-acoustic wave in the resonant ME heterostructures. To analyze the impact of the inner radius variations in the performance of the ME antenna, we employed COMSOL Multiphysics V6.0 to carry out a comprehensive Multiphysics field analysis on ME antennas with distinct inner radii. Initially, we conducted an S-parameter analysis to obtain the resonance frequency of ME antennas with varying inner radii. Subsequently, we analyzed the average stress in the magnetic layer at the resonance frequency. Finally, we utilized the piezoelectric and magnetic fields and EM-wave frequency-domain modules to simulate the entire ME antenna process and obtain its radiation pattern. The operational frequency of the ME antenna discussed in this article is 170 MHz. The piezoelectric layer employs AlN, while the magnetic layer is composed of FeGaB. The equivalent wave speed of the M-P laminated structure is given as [22]

(1) $$\begin{array}{c} v_{eq}=\sqrt{\frac{\frac{n_{m}}{s_{B11}}+\frac{n_{p}}{s_{E11}}}{n_{m}\rho_{m}+n_{p}\sigma_{p}}}\\ s_{B}={\left(c_{H}+e_{H}^{T}\mu_{s}^{-1}e_{H}\right)}^{-1}\\ s_{E}=c_{E}^{-1} \end{array}$$

where $s_{B}$ and $s_{E}$ are the mechanical compliance constants of the M phase and P phase, respectively; $n_{m}$ and $n_{p}$ are the volume ratios of the M phase and P phase, respectively. The simulation is validated against the existing simulation performed on FeGaB/AlN [7]. It can be observed that the proposed scheme is able to capture the S11 curve and the corresponding resonance frequency in the illustration in Figure 2. The two sets of results are in very close agreement, indicating that the simulation may be valid for simulating the electromechanical characteristics. The performance of the ME antenna is studied in the following subsection in which we present the stress–strain analysis, S11 curve, and far-field radiation of the ME antenna.

**Figure 3:**
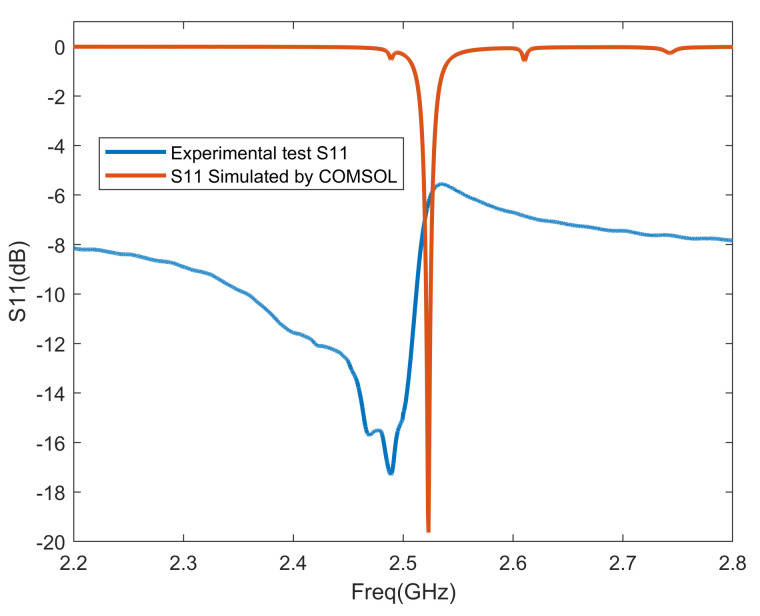
Comparison of simulated S11 and experimental S11 of magnetoelectric antenna.

Additionally, since the magnetoelectric antenna was not fabricated this time, we validated the accuracy of our simulation method using both the simulation and experimental data from a previously designed 2.45 GHz magnetoelectric antenna, as referenced in [10]. The simulation method employed in this paper is identical to the one used in the previous study to simulate the electromechanical characteristics of the magnetoelectric antenna. The comparison between the simulated S11 data and the experimental data is shown in the Figure 3:

It can be observed that there is a slight difference between the resonance point of the simulated ME antenna and that of the actual tested antenna; however, both are essentially close to the designed resonance point. The primary reasons for this discrepancy include the precision of the processing equipment, environmental factors, and the inconsistent quality of the prepared film during fabrication. Additionally, there are some variations between the parameters of the materials used in the simulation and those of the actual processed materials. These factors contribute to the differences between the simulated S11 data and the actual test data of the ME antenna.

We consider a two-layer ME-laminated composite structure with R = 484 μm, tm = 20 μm (thickness of magnetic layer), and tp = 20 μm (thickness of piezoelectric layer). The piezoelectric material is AlN. The magnetostrictive (Terfenol-D) material parameters are obtained from [12]. The piezoelectric module in COMSOL Multiphysics was used for the electromechanical simulation of the ME antenna. Figure 2 shows the simulated reflection coefficient (S11) of the ME antenna, showing an estimated electromechanical resonance frequency of 169 MHz.

## 3. Result and Discussion

**Figure 4:**
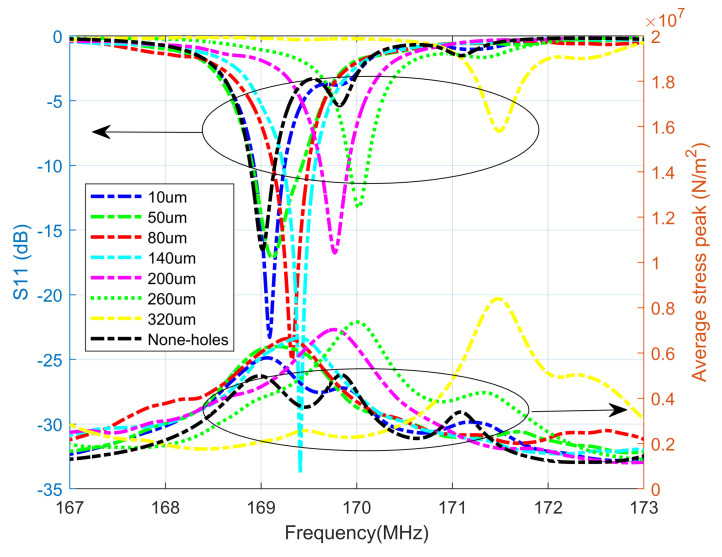
S11 and the average stress peak of the magnetosphere at different radii.

Firstly, we performed simulations for the S11 parameters of each structure with inner radii of 10 μm, 50 μm, 80 μm, 140 μm, 200 μm, 260 μm, and 320 μm, respectively. Secondly, we obtained the average stress values of the magnetic layer for various inner diameters, while considering the frequency variation. The simulation data are visualized in Figure 4.

As shown in Figure 4, different radii will affect the resonance frequency and −10 dB bandwidth of the ME antenna. Except for the resonance frequency with a radius of 320 μm at 171.5 MHz, the resonance points of other ME antennas are ultimately between 169 and 170 MHz.

Meanwhile, we can see that the S11 resonance point corresponds to the maximum von Mises peak stress in the magnetic layer, verifying that the ME antenna is in electromechanical resonance at this frequency point. The bandwidth data are shown in Table 1.

It can be found that, compared to circular ME antennas, as the inner radius increases, the bandwidth first increases and then decreases. When the radius is 50 μm, the bandwidth reaches its maximum value, which is 0.5212 MHz, with an increase of 104%. It is obvious that the stress concentration effect generated by the inner radius is effective for the bandwidth of the ME antenna.

Furthermore, we compared the existing papers related to bandwidth and gain indicators in the literature and listed in Table 2.

As can be seen from the table, compared with the literature that gives the size, bandwidth and gain data of ME antennas, the size of the ME antenna in this article is the smallest: 0.25 mm^2^. Except for the third antenna, the rest are ME antennas based on thin-film bulk-acoustic resonators, and our antenna frequency is currently the lowest.

**Figure 5:**
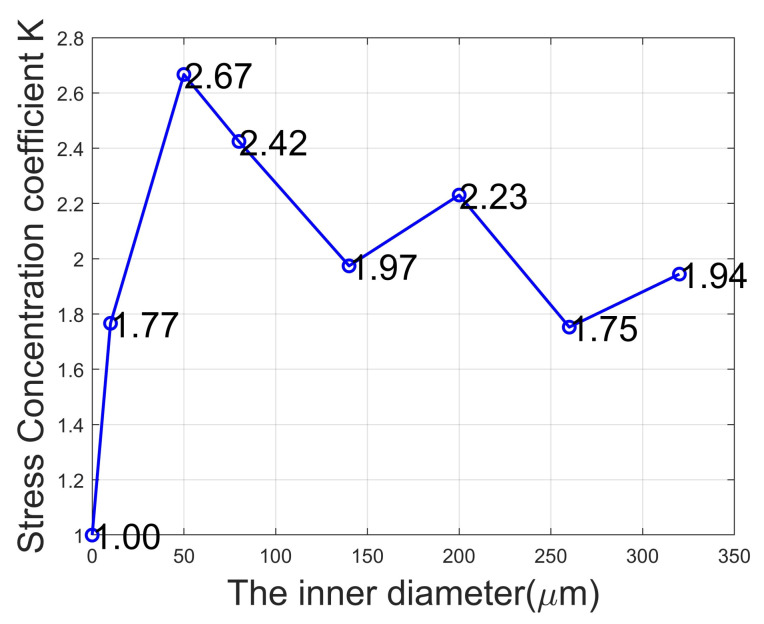
Stress concentration coefficient magnetic layer.

Furthermore, we use COMSOL software to observe the average stress magnetic layers with different radii. Here, we follow the concept of stress concentration and set a stress concentration coefficient K as follows:

(2) $$K=\frac{\sigma_{r}}{\sigma_{0}}$$

where $\sigma_{r}$ represents the average stress of the magnetic layer with an inner radius of r, and $\sigma_{0}$ is the average stress of the magnetic layer without an inner radius. We use COMSOL Multiphysics to extract the average stress of the magnetic layer under different inner radii. For the convenience of observation, we draw a line chart to display the data. From Figure 5, it can be seen that when the inner radius is 50 μm, the average stress of the magnetic layer is the highest, and compared to the circular ME antenna, the stress concentration coefficient is 2.67.

**Figure 6:**
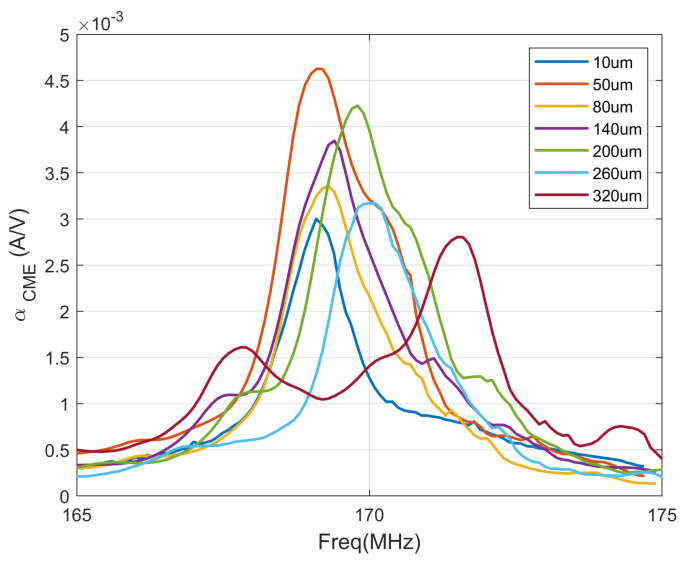
Variation of the converse ME coefficient and the inner diameter of the ring ME antenna.

Given the critical role of the converse ME coefficient in the radiation performance of ME antennas, we conducted simulations to analyze the effects of varying the ring radius on this coefficient. The expression for the converse ME coefficient is given by: $\alpha_{CME}=\left|H_{x}/E_{x}\right|$. This coefficient is commonly used to characterize the ME coupling in a ME resonator, where Hx represents the average AC magnetic field induced in the magnetic layer, and Ez denotes the average electric field in the piezoelectric phase. Furthermore, we modeled the near-field radiation of the ME antenna by surrounding it with an air domain of radius r=500μm. Applying a 1 mV AC voltage to the piezoelectric layer induces alternating stress in the layer. This stress is transferred to the magnetic layer, generating the piezomagnetic effect. The simulation incorporates the piezoelectric effect of the piezoelectric layer using the AC/DC and solid mechanics modules within the structural mechanics module, while the magnetostrictive layer is modeled using the magnetic field and structural mechanics modules in the AC/DC module. We simulated the corresponding converse MEcoefficients at various radii, and the results are shown in Figure 6:

We observed that the relationship between the inverse ME coefficient and the radius initially increases and then decreases as the radius increases. The inverse ME coefficient reaches its peak at 50 µm. This finding aligns with the law of the stress concentration coefficient, indicating that higher average stress in the magnetic layer results in stronger radiation from the ME antenna.

By using the one-dimensional ME-laminated-structure analytical model given in the Appendix A, the expression for the radiation power of a two-layer ME antenna is obtained as follows:

(3) $$P_{rad}=\frac{A\omega^{2}d^{2}T_{0}^{2}d_{H}^{2}}{16\eta_{0}}$$

**Figure 7:**
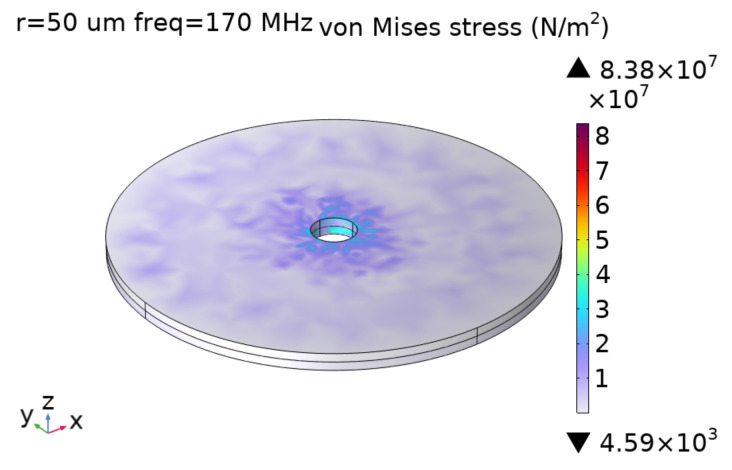
The results of shape optimization.

From this expression, it can be reflected that the radiation power of an ME antenna is not only related to the average stress of the magnetic layer of the ME antenna, but also to the radiation area, the operating frequency and material parameters. For a ring-shaped ME antenna, the expression for its radiation area *A* is $A=\pi (R^{2}-r^{2})$, where *R* is the outer diameter of the ME antenna and *r* is the inner diameter. Based on this, we establish an optimization model targeting the radiation performance of ME antennas:

(4) $$\left\{\begin{array}{c} Find:r\in r_{1},r_{2},r_{3}\ldots ,r_{n}\\ Max:P_{rad}=\frac{\pi \left(R^{2}-r^{2}\right)\omega^{2}d^{2}T_{0}^{2}d_{H}^{2}}{16\eta_{0}}\\ s.t.0<r<R\\ R=const,u_{R}=0 \end{array}\right.$$

where ω is the circular frequency at which the ME antenna operates, *d* is the total thickness of the ME antenna, $d_{H}$ is the piezomagnetic coefficient, and $\eta_{0}$ is the wave impedance in vacuum. Then, we use COMSOL for shape optimization, and the optimization results are shown in Figure 7.

Lastly, we perform a direct radiation simulation of the ME antenna using COMSOL Multiphysics. We use four modules: electrostatic, solid mechanics, magnetic field, and EM waves to perform the simulation in the following steps:

(1) The behavior of the ME antenna’s near field is simulated using solid mechanics, electrostatics, and magnetic fields. Specifically, solid mechanics and electrostatics are applied to model the piezoelectric phase, while solid mechanics and magnetic fields are employed for the magnetostrictive phase.
(2) The far field of the ME antenna is simulated using the EM wave module. In the vicinity of the ME antenna, a sphere is employed. Through the principle of surface equivalence, the equivalent magnetic current and equivalent current of the sphere are derived. These equivalent values are then utilized as the new radiation source for calculating the far-field radiation.

**Figure 8:**
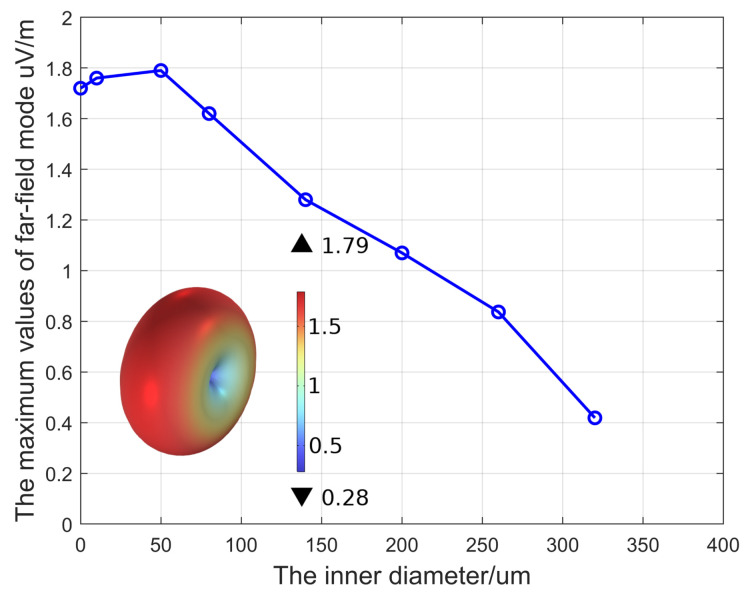
Maximum values of far-field modes under different inner radii.

As shown in the inset of Figure 8, the radiation pattern of an ME antenna is similar to that of a dipole. Here, we only present the radiation pattern of the ME antenna with an inner radius of 50 μm; its far-field mode maximum value is 1.79 μV/m and the minimum value is 0.28 μV/m. The radiation patterns of other ME antennas are also similar to that of a dipole, with the difference being the maximum value. Here, we list the maximum values of far-field modes corresponding to different inner radius in Table 3. The far-field mode of the electric field is a physical quantity related to distance in the COMSOL software, which can reflect the antenna radiation capability. Under the spherical air domain simulation, ME antennas with different inner diameters have different maximum values of far-field modes, reflecting changes in the antenna’s radiation ability as the inner diameter changes.

From Figure 6, it can be clearly seen that as the inner diameter increases, the radiation capability first increases and then weakens. When the inner radius is 50 μm, the maximum far-field mode reaches its maximum value, which is 1.79 uV/m. Regarding the calculation method of the antenna gain in this paper, the directivity coefficient of the ME antenna obtained by COMSOL simulation is D = 1.51, and based on the radiation efficiency η given in Ref. [7] as η=0.28%, the gain of the antenna can be obtained: Gain = −21.59 dBi.

## 4. Conclusions

In summary, the primary objective of this study is to enhance the bandwidth and radiation performance of ME antennas through structural optimization design. It has been determined that the ring-shaped ME antenna exhibits a comparatively higher average stress in its magnetic layer, which theoretically enables the generation of greater radiation power. To corroborate this finding, a comprehensive full-process simulation of the ME antenna was conducted, and the results obtained were consistent with the aforementioned conclusions.

This paper investigates the full-field coupling model of ME antennas and explores the potential of enhancing their bandwidth and radiation performance through structural optimization. These findings offer a promising avenue for designing ME antennas that are practically applicable in engineering applications.

## Figures and Tables

**Table 1:** Bandwidth at different inner radii.

The Radius (μm)	−10 dB Bandwidth (MHz)	Fractional Bandwidth (FBW)
0	0.2533	0.1498%
10	0.3211	0.1899%
50	0.5212	0.3082%
80	0.3717	0.2195%
140	0.3324	0.1962%
200	0.2888	0.1702%
260	0.1896	0.1115%
320	0	0%

**Table 2:** Information of ME antenna bandwidth, gain and dimensions as reported in the literature.

Paper	Bandwidth	Resonant Frequency (f0)	Dimension	Gain
Ref. [16]	−3 dB 21 MHz	800 MHz	0.49 mm^2^	None
Ref. [9]	−3 dB 42 MHz	1.575 GHz	3 mm × 3 mm	−19.4 dBi
Ref. [23]	−3 dB 725 Hz + 1179 Hz	36 kHz	50 mm × 6 mm	None
Ref. [7]	None	2.53 GHz	0.7 mm × 0.8 mm	−18 dBi
This paper	−10 dB 0.5212 MHz	170 MHz	0.5 mm × 0.5 mm	−21.59 dBi

**Table 3:** Maximum value of far-field modes under different inner radii.

**The radius (μm)**	0	10	50	80	140	200	260	320
**Maximum value (μV/m)**	1.72	1.76	1.79	1.62	1.28	1.07	0.84	0.44

## Data Availability

The data that support the findings of this study are available from the corresponding author upon reasonable request.

## Abbreviations

The following abbreviations are used in this manuscript:

ME	magnetoelectric
EM	electromagnetic
FDTD	finite difference time-domain
HBAR	high-overtone bulk-acoustic resonator
FPA	floating potential structure

## Appendix A

The radiation capability of the antenna can usually be described by the radiation *Q* factor. The *Q* factor represents the ratio of the average stored energy in the antenna structure to the radiation power per cycle:

(A1) $$Q=\omega \frac{W_{T}}{P_{rad}}$$

The potential energy in the ME antenna is equal to the sum of the potential energy in the piezoelectric and the piezomagnetic layers:

(A2) $$W_{PE}=\frac{1}{2}\;\int \int \int \;S\cdot Tdv+\frac{1}{2}\;\int \int \int \;D\cdot Edv$$

(A3) $$W_{PM}=\frac{1}{2}\;\int \int \int \;S\cdot Tdv+\frac{1}{2}\;\int \int \int \;B\cdot Hdv$$

The dynamic strain is transferred from the piezoelectric layer to the magnetic layer, and then the dynamic strain excites a uniform dynamic magnetic flux within the magnetic layer. According to Faraday’s law, this dynamic magnetic flux generates d dynamic electric field, which varies linearly along the thickness dimension until it reaches above the surface of the magnetic layer where an aperture electric field is formed. Denoting the aperture electric field by, the average radiated power of the EM wave is thus calculated to be

(A4) $$P_{rad}=\frac{1}{2\eta_{0}}\;\int \int \;{\left|E_{0}\right|}^{2}ds$$

Using Faraday’s law E=ωh|B|, where *h* is the thickness of the magnetic layer, Formula (8) can be written as

(A5) $$P_{rad}=\frac{\omega^{2}h^{2}d_{H}^{2}}{2\eta_{0}}\;\int \int \;{\left|T_{H}\right|}^{2}ds$$

If the ME antenna has a two-layer structure, we can obtain a unique *Q* factor expression:

(A6) $$Q=\frac{A\omega^{2}d^{2}T_{0}^{2}d_{H}^{2}}{16\eta_{0}}$$
